# Applications of Extended Reality in Ophthalmology: Systematic Review

**DOI:** 10.2196/24152

**Published:** 2021-08-19

**Authors:** Chee Wui Ong, Marcus Chun Jin Tan, Michael Lam, Victor Teck Chang Koh

**Affiliations:** 1 Yong Loo Lin School of Medicine National University of Singapore Singapore Singapore; 2 Department of Ophthalmology National University Hospital Singapore Singapore; 3 Department of Ophthalmology Ng Teng Fong General Hospital Singapore Singapore; 4 Department of Ophthalmology Yong Loo Lin School of Medicine National University of Singapore Singapore Singapore

**Keywords:** extended reality, virtual reality, augmented reality, mixed reality, ophthalmology, ophthalmic

## Abstract

**Background:**

Virtual reality, augmented reality, and mixed reality make use of a variety of different software and hardware, but they share three main characteristics: immersion, presence, and interaction. The umbrella term for technologies with these characteristics is extended reality. The ability of extended reality to create environments that are otherwise impossible in the real world has practical implications in the medical discipline. In ophthalmology, virtual reality simulators have become increasingly popular as tools for surgical education. Recent developments have also explored diagnostic and therapeutic uses in ophthalmology.

**Objective:**

This systematic review aims to identify and investigate the utility of extended reality in ophthalmic education, diagnostics, and therapeutics.

**Methods:**

A literature search was conducted using PubMed, Embase, and Cochrane Register of Controlled Trials. Publications from January 1, 1956 to April 15, 2020 were included. Inclusion criteria were studies evaluating the use of extended reality in ophthalmic education, diagnostics, and therapeutics. Eligible studies were evaluated using the Oxford Centre for Evidence-Based Medicine levels of evidence. Relevant studies were also evaluated using a validity framework. Findings and relevant data from the studies were extracted, evaluated, and compared to determine the utility of extended reality in ophthalmology.

**Results:**

We identified 12,490 unique records in our literature search; 87 met final eligibility criteria, comprising studies that evaluated the use of extended reality in education (n=54), diagnostics (n=5), and therapeutics (n=28). Of these, 79 studies (91%) achieved evidence levels in the range 2b to 4, indicating poor quality. Only 2 (9%) out of 22 relevant studies addressed all 5 sources of validity evidence. In education, we found that ophthalmic surgical simulators demonstrated efficacy and validity in improving surgical performance and reducing complication rates. Ophthalmoscopy simulators demonstrated efficacy and validity evidence in improving ophthalmoscopy skills in the clinical setting. In diagnostics, studies demonstrated proof-of-concept in presenting ocular imaging data on extended reality platforms and validity in assessing the function of patients with ophthalmic diseases. In therapeutics, heads-up surgical systems had similar complication rates, procedural success rates, and outcomes in comparison with conventional ophthalmic surgery.

**Conclusions:**

Extended reality has promising areas of application in ophthalmology, but additional high-quality comparative studies are needed to assess their roles among incumbent methods of ophthalmic education, diagnostics, and therapeutics.

## Introduction

The rapid development of extended reality technologies has necessitated recent efforts to define and draw lines between new concepts and subgroups of extended reality applications [1]. Virtual reality has been defined as one in which our natural surroundings are completely replaced with a 3D computer-generated environment via wearable screens in the form of head-mounted displays [2]. Augmented reality is a superimposition of computer-generated content with limited interactivity onto our visible surroundings. Mixed reality is similar to augmented reality, except that the user is able to interact vividly with computer-generated content [1]. Mixed reality can be considered an amalgamation of the features of both virtual reality and augmented reality, as both highly interactive computer-generated objects and the real physical world are integrated to dynamically coexist within a single display [3,4]. Whereas virtual reality, augmented reality, and mixed reality make use of a variety of different software and hardware, these extended reality technologies share 3 main characteristics: immersion, presence, and interaction [2,5]. *Immersion* refers to a perception of physical existence within the extended reality environment, *presence* describes the perception of connection to the environment, whereas *interaction* is the ability to act and receive feedback within the environment [2].

In medicine, the nascent influence of extended reality is prevalent. Virtual reality platforms have been designed to teach foundational subjects, such as human anatomy [6,7], and train surgeons in complex surgical procedures [8-11]. Augmented and mixed reality offer methods of visualizing intraoperative procedures and diagnostic images with devices, such as Google Glass (Google Inc) or Microsoft HoloLens (Microsoft Inc), that have the potential to improve procedure safety and success [12-14]. The ability of virtual reality to distract patients from the physical environment also offers therapeutic approaches for rehabilitation and for treating pain or psychiatric disorders [15-17]. Likewise, ophthalmology has seen a growing influence of extended reality. Ophthalmic graduate medical education in the United States has seen an increase in the use of virtual eye surgery simulators, from 23% in 2010 to 73% in 2018 [18,19]. Extended reality technologies have also been explored as a method of therapy in ophthalmic diseases such as amblyopia and visual field defects [20,21]. Although the versatility of extended reality platforms can influence the practice of ophthalmology, health care providers should be well informed of the benefits and limitations of such technologies. This will allow evidence-based decision making when adopting nascent methods of ophthalmic education, diagnosis, and treatment. The focus of this review was to systematically evaluate current evidence of the efficacy, validity, and utility of the application of extended reality in ophthalmic education, diagnostics, and therapeutics.

## Methods

### Eligibility Criteria

We included studies evaluating the use of extended reality for ophthalmic applications in education, diagnostics, and therapeutics for eye care professionals and ophthalmic patients. All study designs were included with the exception of systematic reviews, case reports, and case series with ≤3 patients. Non-English publications and publications on the technical engineering of extended reality were excluded.

### Search Methods

Three databases served as the source of our search—PubMed MEDLINE, Embase, and Cochrane Register of Controlled Trials. Search terms included “Virtual Reality,” “Augmented Reality,” “Mixed Reality,” “Simulation,” “Simulated,” “3D,” “Ophthalmology,” “Ophthalmic,” and “Eye.” The search was performed on April 15, 2020. Publications from January 1, 1956 to April 2020 were searched without language or publication-type restrictions. References in studies meeting the eligibility criteria were searched to identify additional eligible studies. EndNote X9 (2020; Clarivate Analytics) was used to manage all identified publications and remove duplicates (Multimedia Appendix 1). Search results were recorded according to PRISMA (Preferred Reporting Items for Systematic Reviews and Meta-analyses) guidelines [22].

### Study Selection

Two authors (CWO and MCJT) read all titles returned by the search. All abstracts of relevant titles and full texts of the relevant abstracts were read by the same authors to evaluate eligibility. Any uncertainties was resolved by discussion among all authors.

### Data Collection and Analysis

For each study that met eligibility criteria, the quality of study was evaluated using Oxford Centre for Evidence-Based Medicine (OCEBM) levels of evidence [23].

Information from each study was extracted, including aim, design, population, sample size, extended reality technology type, application, outcomes, and findings.

A number of eligible studies investigated the use of extended reality educational training simulators as training and assessment tools. Evidence of validity should be used to support the appropriateness of interpretation of results from assessments of performance using these simulators [24,25]. Validation is critical to be able to trust the results of a given education tool, and educators need evidence of validity to identify the appropriate assessment tool to meet specific educational needs with finite resources [26]. We chose a contemporary model of validity [24], comprising 5 sources of validity evidence—Content, Response process, Internal structure, Relationship to other variables, and Consequences [25,27] (Multimedia Appendix 2), to evaluate the extent to which the validity of these simulator-based assessments had been established by evidence. Due to a high degree of heterogeneity between studies, quantitative statistical analysis was not conducted.

## Results

### General

**Figure 1:**
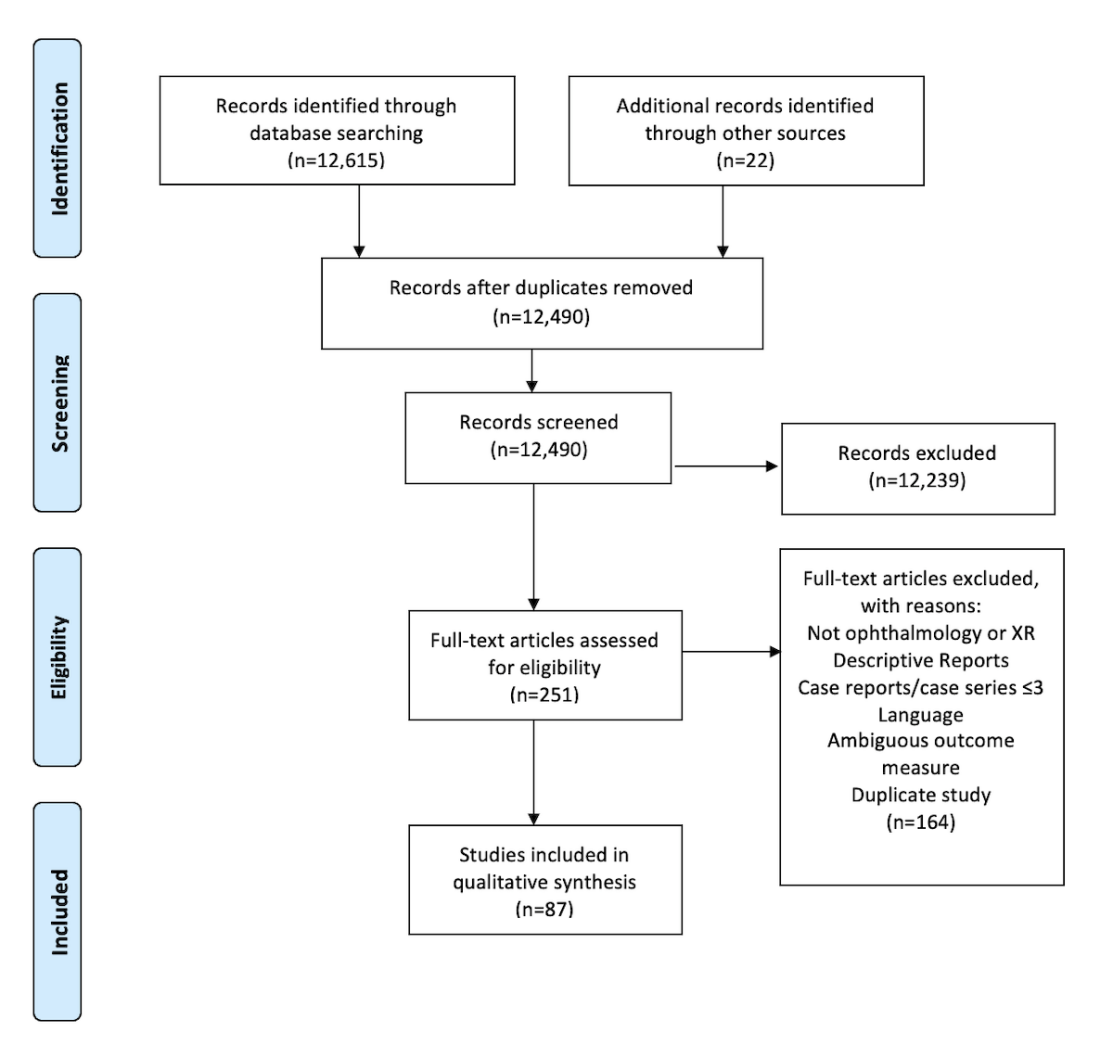
Flow Diagram showing inclusion process for identified records. XR: extended reality.

A total of 12,490 unique records were identified. After screening by title and abstract, 251 full-text publications were retrieved for assessment for final eligibility. Of these, 164 were excluded, and 87 studies met the final eligibility criteria (Figure 1). Of these, 54 were relevant to the use of extended reality in education, 5 were relevant to the use of extended reality in diagnostics, and 28 were relevant to the use of extended reality in therapeutics.

### Education

#### Overview

Applications of extended reality in education included surgical simulators (46/54), ophthalmoscopy simulators (6/54), and optometry training simulators (2/54), with medical students, optometry students, trainee, or trained ophthalmologists as participants.

#### Surgical Simulators

Of 46 studies evaluating surgical simulators, the EyeSi surgical simulator (VR Magic) was most commonly used (n=38). Others included MicroVisTouch (ImmersiveTouch) (n=1), PixEye Ophthalmic Simulator (SimEdge SA) (n=1), and 6 self-designed simulators. The most common surgical procedure simulated in these studies was cataract surgery (n=36), followed by vitreoretinal procedures (n=9), laser trabeculoplasty (n=1), and corneal laceration repair (n=1).

Of the 46 studies, 24 were studies that evaluated the efficacy of surgical simulators using evaluations of surgical performance on real patients, objective assessments by subspecialty experts, or simulator-based metrics as outcome measures. There were 14 [28-47] studies that used surgical performance on real patients as outcome measures, of which 4 were randomized trials [48-51] (Table 1); these randomized trials compared the use of virtual reality ophthalmic surgical simulators with conventional methods of surgical training. Objective assessment metrics of participants’ surgical performance on real patients were evaluated by subspecialty experts. All studies [48,50,51], except one [49], showed that simulator-training resulted in surgical performance significantly superior to that of conventional training methods in terms of quality, time efficiency, efficacy, or complication rates. In particular, Deuchler et al [50] found that warm-up simulation training improved performance for surgeons who had not operated for a significant period of time. Daly et al [49] showed that residents who underwent EyeSi training were significantly slower at performing their first continuous curvilinear capsulorhexis than participants who underwent wet-lab training but achieved similar surgical performance scores.

**Table 1:** Randomized trials evaluating efficacy of surgical simulators in improving surgical performance on real patients.

Study	Design (OCEBM^a^ level)	Intervention group training	Control group training	Simulated task	Population	Findings
Peugnet (1998) [48]	Randomized trial (2b)	Laser photocoagulation simulator (n=5)	Real patients (n=4)	Retinal photo-coagulation	Eye residents	Simulator training was significantly more time efficient (time efficiency index of 0.59 vs 0.28, *P*<.05), and resulted in a trend of greater photocoagulation efficiency (duration/impact index of 0.040 vs 0.028)
Daly (2013) [49]	Randomized trial (2b)	EyeSi (n=11)	Wet lab (n=10)	Continuous curvilinear capsulorhexis	Eye residents	Wet-lab trained residents significantly faster than EyeSi-trained residents (*P*=.038)
Deuchler (2016) [50]	Randomized trial (1b)	EyeSi warm-up (n=9)	No warm-up (n=12)	Pars plana vitrectomy	Surgeries by vitreoretinal surgeons	EyeSi warm-up improved surgical performance significantly (GRASIS^b^ score of 1.0 vs 0.5, *P*=.0302)
Alwadani (2012) [51]	Randomized trial (2b)	PixEyes simulator (n=24)	Didactic, wet lab (n=23)	Argon laser trabeculoplasty	Medical students	VR-trained students had lower rates of inadvertent corneal/iris burns (4.5% vs 34.0%, *P*=.01), delivery misses (8% vs 55%, *P*=.001), overtreatment and undertreatment (7% vs 46%, *P*=.015)

^a^OCEBM: Oxford Centre for Evidence-Based Medicine.

There were 10 nonrandomized studies [28-37] that evaluated the EyeSi simulator for cataract surgery training (Table 2). Thomsen et al [28] and la Cour et al [29] demonstrated statistically significant improved Objective Structured Assessment of Cataract Surgical Skill Scores (OSACSS) in novice surgeons after training with the EyeSi cataract module. Roohipoor et al [30] found significant correlations between residents’ EyeSi simulator-based scores and their eventual surgery count and Global Rating Assessment of Skills in Intraocular Surgery scores. The other 7 studies [31-37] investigated the relationship between EyeSi simulator use and complication rates in cataract surgery on real patients, of which 6 studies [31-36] showed that the use of the EyeSi simulator was associated with reduced complication rates. McCannel et al [37] found that EyeSi capsulorhexis training was not associated with lower vitreous loss rates overall but was associated with higher nonerrant continuous curvilinear capsulorhexis-associated vitreous loss.

**Table 2:** Nonrandomized trials evaluating efficacy of surgical simulators in improving surgical performance on real patients.

Study	Design (OCEBM^a^ level)	Comparison or control group	Outcome measure	Participants and cataract surgeries, (n)	Findings
Thomsen (2017a) [28]	Cohort (2b)	Before EyeSi use	OSACSS^b^	Cataract surgeons (19)	Novices and less-experienced surgeons showed significant improvements in the operating room (32% and 38% improvement, *P*=.008 and *P*=.018 respectively) after EyeSi training
La Cour (2019) [29]	Cohort (2b)	Before EyeSi use	OSACSS	Cataract surgeons (19)	EyeSi training resulted in significantly improved surgical performance in less-experienced surgeons. Skill-transfer between modules was not demonstrable
Roohipoor (2017) [30]	Cohort (2b)	N/A^c^	GRASIS^d^	Ophthalmology residents (30)	Significant correlations between residents’ EyeSi simulator-based scores and their eventual surgery count and GRASIS scores
Belyea (2011) [31]	Cohort (3b)	No EyeSi use	Phaco time, percentage power, complications	Surgeries by ophthalmology residents (592)	EyeSi training resulted in significantly lower procedure duration (*P*=.002), percentage power (*P*=.001), and nonsignificantly fewer intraoperative complications
Pokroy (2013) [32]	Cohort (2c)	No EyeSi use	Incidence of posterior capsule tears, operation duration	Surgeries by ophthalmology residents (1000)	EyeSi training resulted in nonsignificantly fewer posterior capsule tears and shorter learning curves
Ferris (2020) [33]	Cohort (2b)	No EyeSi access	Posterior capsule rupture rates	Surgeries by ophthalmology residents (17831)	Residents with EyeSi access had a significant reduction in posterior capsule rupture rates (4.2% vs 2.6%, Difference in Proportions 1.5%, 95% CI 0.5-2.6%, *P*=.003). Posterior capsule rupture rates significantly lower after access to EyeSi (3.5% to 2.6%, Difference in proportions 0.9%, 95% CI 0.4-1.5%, *P*=.001)
Lucas (2019) [34]	Cohort (2b)	No EyeSi use	Complication rates	Surgeries by ophthalmology residents (140)	EyeSi training resulted in significantly fewer complications (12.86 vs 27.14%, *P*=.031)
Staropoli (2018) [35]	Cohort (2b)	No EyeSi use	Complication rates	Surgeries by ophthalmology residents (955)	EyeSi training resulted in significantly fewer complications (2.4 vs 5.1%, *P*=.037)
McCannel (2013) [36]	Case series (4)	Reduced EyeSi use	Errant continuous curvilinear capsulorhexis rates	Surgeries by ophthalmology residents (1037)	EyeSi training resulted in significantly lower errant continuous curvilinear capsulorhexis rates (5.0 vs 15.7%, *P*<.001)
McCannel (2017) [37]	Case series (4)	Reduced EyeSi use	Vitreous loss rates, retained lens material	Surgeries by ophthalmology residents (1037)	EyeSi training was not associated with lower vitreous loss rates or less retained lens material but was associated with higher vitreous loss in nonerrant CCCs^e^

^a^OCEBM: Oxford Centre for Evidence-Based Medicine.

^b^OSACCS: Objective Structured Assessment of Cataract Surgical Skill Score.

^c^N/A: not applicable.

^d^GRASIS: Global Rating Assessment of Skills in Intraocular Surgery.

^e^CCC: continuous curvilinear capsulorhexis.

Seven studies [38-44] investigated the efficacy of the EyeSi surgical simulator (n=6) or a self-made augmented reality microsurgery simulator (n=1) by evaluating participants’ surgical performance on the same simulators using simulator-based metrics (Table 3 and Table 4). Selvander et al [40] used the OSACSS and Objective Structured Assessment of Technical Surgical Skills (OSATS).

Three studies were randomized trials [38-40] (Table 3). Thomsen et al [39] investigated if there could be interprocedural transfer of skills and found that residents with simulated cataract surgery training did not perform significantly better than those without (simulator score with training: mean 381, SD 129 vs simulator score without training: mean 455, SD 82, *P*=.262) at the vitreoretinal surgery module. Selvander et al [40] had a similar aim and found that training on the capsulorhexis or cataract navigation training module on the EyeSi did not significantly improve performance at the other (OSACSS—training: 8, no training: 8, *P*=.64; OSATS score—training: 7, no training: 10, *P*=.52); however, repeated practice with each module significantly improved simulator-based scoring for the respective modules. Bergqvist et al [38] found that medical students who trained with simulated cataract surgery had higher overall simulator scores and fewer complications than those who did not train on the simulator. These trials suggest that simulator training improves performance only for the specific procedure being trained. The other 4 studies [41-44] were prospective cohort studies or case series (Table 4) that demonstrated that extended reality surgical training resulted in significant improvements on subsequent simulator-based performance scores.

**Table 3:** Randomized trials evaluating efficacy of surgical simulators in improving surgical performance as measured by the same simulator.

Study	Design (OCEBM^a^ level)	Intervention group	Control group	Participants	Simulated task
Bergqvist (2014) [38]	Randomized trial (1b)	EyeSi training (n=10)	No EyeSi training (n=10)	Medical students	Cataract surgery
Thomsen (2017b) [39]	Randomized trial (2b)	EyeSi training (n=6)	No EyeSi use (n=6)	Eye residents	Cataract surgery, vitreoretinal surgery
Selvander (2012) [40]	Randomized trial (2b)	EyeSi cataract navigation training first (n=17)	EyeSi capsulorhexis training first (n=18)	Medical students	Capsulorhexis, cataract navigation

^a^OCEBM: Oxford Centre for Evidence-Based Medicine.

**Table 4:** Nonrandomized trials evaluating efficacy of surgical simulators in improving surgical performance as measured by the same simulator.

Study	Design (OCEBM^a^ level)	Surgical simulator	Participants	Simulated task	Findings
Saleh (2013a) [41]	Case series (4)	EyeSi	Eye residents (n=17)	Cataract surgery	EyeSi training resulted in significantly improved scores (*P*<.001) among residents, especially for capsulorhexis and antitremor
Gonzalez-Gonzalez (2016) [42]	Case series (4)	EyeSi	Medical students, eye residents (n=14)	Capsulorhexis	EyeSi training resulted in significantly improved course scores for both dominant (33.4 vs. 46.5; *P*<.05) and nondominant hands (28.9 vs. 47.7; *P*<.001) and faster performance times (*P*<.001)
Bozkurt (2018) [43]	Cohort (2b)	EyeSi	Ophthalmologists (n=16)	Capsulorhexis module	EyeSi training resulted in significantly improved capsulorhexis scores (*P*=.001)
Ropelato (2020) [44]	Case series (4)	Microsoft HoloLens	Unspecified (n=47)	Internal limiting membrane peeling	There was significant improvement in micromanipulation performance scores after simulator-training

^a^OCEBM: Oxford Centre for Evidence-Based Medicine.

Three randomized trials investigated the effect of extended reality surgical simulator training on surgical performance in the wet lab (Table 5). Surgical performance was assessed using objective outcomes. Feldman et al [46] and Feudner et al [47] showed that training on the EyeSi simulator significantly improved wet-lab performance for corneal laceration repair and capsulorhexis, respectively, while Jonas et al [45] showed that training on a self-made virtual reality simulator improved wet-lab performance for pars plana vitrectomies.

**Table 5:** Studies evaluating efficacy of surgical simulators in improving surgical performance in the wet lab.

Study	Design (OCEBM^a^ level)	Intervention group	Control group	Participants	Outcome measure tool
Jonas (2003) [45]	Randomized trial (2b)	Simulator training (n=7)	No simulator training (n=7)	Medical students, eye residents	Amount of vitreous removed, retinal lacerations, residual retinal detachment, duration
Feldman (2007) [46]	Randomized trial (2b)	EyeSi training (n=8)	No EyeSi training (n=8)	Medical students	Corneal Laceration Repair Assessment
Feudner (2009) [47]	Randomized trial (1b)	EyeSi training (n=31)	No EyeSi training (n=31)	Eye residents	Scoring based on capsulorhexis video

^a^OCEBM: Oxford Centre for Evidence-Based Medicine.

Of 46 studies, 20 studies evaluated the validity of surgical simulator-based assessments (Multimedia Appendix 3). Most validity studies achieved an OCEBM level of evidence of 2b, corresponding to exploratory cohort studies with good reference standards. The most common source of validity evidence was *Relationship with other variables*, addressed in 19 of 20 studies (95%). Studies achieved this by statistically evaluating the relationship between surgical performance on the simulator and participants’ levels of expertise. *Content validity* was addressed in 18 studies (90%), *Response process* was addressed in 9 studies (45%), *Internal structure* was addressed in 5 studies (25%), and *Consequences* was addressed in 2 studies (10%). Only 2 of 20 (10%) studies addressed all 5 sources.

Of these 20 studies, 12 assessed surgical performance using simulator-based scoring only [39,43,52-61], 6 studies compared simulator-based scores with video-based scoring (OSACSS, OSATS, or motion-tracking software) [62-67], and 2 studies used video-based scoring only [40,68].

For the EyeSi surgical simulator, most studies found that the surgical performance of experienced participants was significantly better than that of less-experienced participants. Sikder et al [52] found that intervening surgical experience significantly improved capsulorhexis performance on the MicroVisTouch cataract surgery simulator. Lam et al [62] showed that in a self-made phacoemulsification simulator, more experienced participants attained significantly higher scores in all main procedures and completed tasks significantly faster.

Five studies [69-73] assessed the perception of ophthalmologists and medical students toward surgical simulators using user-reported outcome measures. These studies achieved OCEBM evidence levels of 4 (n=4) and 2b (n=1). Users found the EyeSi and a novel virtual reality continuous curvilinear capsulorhexis simulator to be useful in improving surgical skill, confidence, and understanding, while providing a safe and realistic alternative for training.

#### Ophthalmoscopy Simulators

Six studies [74-79] evaluated the use of extended reality as a tool for education in ophthalmoscopy. Simulators used were the EyeSi Augmented Reality Direct (n=1) and Binocular Indirect (n=3) ophthalmoscopy simulators, and 2 novel self-made direct ophthalmoscopy simulators comprising the HTC Vive Virtual Reality-Head-Mounted Display (n=1) and the RITECH II Virtual Reality-Head-Mounted Display (n=1).

Two randomized trials [74,75], with OCEBM evidence levels 2b, assessed the efficacy of the EyeSi Binocular Indirect Ophthalmoscopy simulator. Both studies showed that participants who trained with the EyeSi Binocular Indirect Ophthalmoscopy simulator performed significantly better than participants who underwent conventional training.

Three studies [75-77], with OCEBM evidence levels of 2b, assessed the validity of the EyeSi Binocular Indirect Ophthalmoscopy simulator (n=2) and the EyeSi Binocular Direct Ophthalmoscopy simulator (n=1) for training and assessment. All studies demonstrated *Relationships with other variables* as a source of validity evidence and found that participants with more experience had significantly higher ophthalmoscopy evaluation scores. *Content validity* was addressed in all studies. Only 1 study [77] addressed *Internal structure* by evaluating internal consistency between simulator modules and evaluated *Consequences* by calculating a pass or fail score.

Two user perception studies [78,79] found that medical students felt that self-assembled virtual reality direct ophthalmoscopy simulators were usable and useful in improving ophthalmoscopy skills.

#### Optometry Training Simulators

Two studies [80,81] evaluated the preliminary user experience of an augmented reality optometry simulator comprising a head-mounted display, a slit-lamp instrument, and a simulated eye, which allowed the simulation of optometry training tasks. User studies involving undergraduate optometry students showed that the simulator was feasible in simulating foreign body removal as a training task with a high level of user satisfaction.

### Diagnostics

#### Overview

Five studies evaluated the use of extended reality for the production of immersive and interactive content for diagnostic applications. Two studies evaluated the use of extended reality to display ocular imaging data [82,83], and 3 studies [84-86] evaluated the validity of extended reality as a simulation tool for the functional assessment of patients with ophthalmic diseases.

#### Ocular Imaging

Two case series, which achieved OCEBM evidence levels of 4, evaluated the presentation of ocular imaging modalities in virtual reality and augmented reality environments.

Maloca et al [82] tested the feasibility of displaying optical coherence tomography images in a virtual reality environment with a virtual reality-head-mounted display. A user perception survey involving 57 participants found it to be well tolerated with minimal side-effects. Berger et al [83] demonstrated feasibility for a method of direct overlay of photographic and angiographic fundus images onto a real-time slit lamp fundus view in 5 participants.

#### Simulators for Functional Assessment

Three studies evaluated the use of extended reality simulators for the functional assessment of patients with ophthalmic diseases. The studies achieved OCEBM levels of evidence of 4.

Goh et al [84] trialed the use of the Virtual Reality Glaucoma Visual Function Test with a smartphone paired with the Google Cardboard head-mounted display to assess the visual function of glaucoma patients and found that stationary test person scores demonstrated criterion and convergent validity, corresponding to *Relationship with other variables*.

Ungewiss et al [85] compared the assessment of driving performance in a driving simulator with that in a real vehicle in patients with glaucoma (n=10), hemianopia (n=10), and normal controls (n=20) and found that patients with hemianopic and glaucoma performed worse than healthy controls on the driving simulator, demonstrating *Relationship with other variables* as a source of validity evidence.

Jones et al [86] evaluated the use of a head-mounted display to simulate visual impairment in glaucoma using virtual reality and augmented reality and found it able to replicate and objectively quantify functional impairments associated with visual impairments. When the simulated visual field loss was inferior, impairments were noted to be significantly greater than those noted when the simulated visual field loss was superior, which was consistent with previous experiences of real patients with glaucoma [87-89].

### Therapeutics

#### Overview

A total of 28 studies evaluated the use of extended reality in therapeutics. These studies evaluated heads-up surgery (n=21), binocular treatment of amblyopia (n=2), functional improvement for the visually impaired (n=4), and an aid for achromatopsia (n=1).

#### Heads-up Surgery

Heads-up surgery involves the use of a 3D camera to capture images from a stereomicroscope for presentation on a 3D display. Of 21 studies [90-110], the most common heads-up surgical system evaluated was NGENUITY 3D (Alcon Laboratories) (n=12), followed by TrueVision 3D HD System (TrueVision Systems Inc) (n=2), TRENION 3D HD (Carl Zeiss Meditec) (n=1), MKC-700HD and CFA-3DL1 (Ikegami) (n=1), Digital Microsurgical Workstation (3D Vision Systems) (n=1), TIPCAM 1S 3D ORL endoscope (Karl Storz) (n=1). Surgical procedures included vitreoretinal procedures (n=17), cataract surgery (n=5), scleral buckle (n=1), and endoscopic lacrimal surgery (n=1).

Six studies had OCEBM evidence levels of 2b, corresponding to randomized trials (n=4) and cohort studies (n=2). There were 15 studies with OCEBM evidence levels of 4, corresponding to case series, case-control studies, or poor-quality cohort studies.

The 4 randomized trials [90-93] demonstrated noninferiority of heads-up surgery in comparison with conventional microscope surgery in postoperative outcomes and complications. Qian et al [90] performed phacoemulsification and intraocular lens implantation and reported no significant difference in mean surgery time, postoperative mean endothelial cell density between conventional surgery (n=10) and heads-up surgery (n=10). Talcott et al [91] performed pars plana vitrectomies and showed that compared with conventional surgery (n=16), heads-up surgery (n=23) significantly increased macular peel time (14.76 minutes vs 11.87 minutes, *P*=.004) but not overall operative time. There was no significant difference in visual acuity (logarithm of the minimum angle of resolution) or change from baseline, and no clinically significant intraoperative complications. Romano et al [92] randomized 50 eyes to the use of an unspecified heads-up surgical system (n=25) and conventional surgery (n=25) for 25-gauge pars plana vitrectomy; there was no significant difference in mean operation duration. Surgeons and observers were significantly more satisfied (*P*<.001) using the heads-up system. Kumar et al [93] randomized 50 patients to macular hole surgery with an unspecified heads-up system (n=25) and conventional macular hole surgery (n=25); there were no significant differences in postoperative visual acuity, macule hole indices, surgical time, total internal limiting membrane peel time, number of flap initiations, and macular hole closure rates. Microscope illumination intensity (heads-up: 100%; conventional: 45%) and endoillumination was significantly lower in heads-up surgery (heads-up: 40%; conventional: 13%).

The other 17 studies [94-110] were case series or cohort studies (Multimedia Appendix 4). They reported experiences with different heads-up surgical systems with various surgical procedures and assessed a few common outcomes with the following findings.

Of studies comparing heads-up surgical systems with conventional surgery, most reported procedural success or success that was not significantly different from that of conventional surgery. There were no significant differences in mean procedural durations, postoperative visual acuity, or improvements in visual acuity. The minimum required endoillumination was lower in heads-up surgery than that in conventional surgery. Zhang et al [94] found a significantly lower mean minimum required endoillumination for heads-up surgery than that for conventional surgery (10% vs 35%, 598.7 vs 1913 lx, *P*<.001). Matsumoto et al [95] operated safely and successfully on 74 eyes with an endoillumination intensity of 3% using a heads-up system. No study reported major perioperative complications or significant differences in complication rates between heads-up surgery and conventional surgery. Users preferred heads-up surgery to conventional surgery.

#### Binocular Treatment of Amblyopia

Two studies [21,111] evaluated the efficacy of the use of extended reality for interactive and immersive binocular treatment for amblyopia. Lee et al [111] randomized 22 children with amblyopia (mean age 8.7 years, SD 1.3) to treatment with virtual reality videogaming on an unspecified virtual reality-head-mounted display (n=7), virtual reality videogaming and Bangerter foil (n=5), or Bangerter foil only (n=10), achieving an OCEBM evidence level of 2b. Two of 7 (29%) of patients in the virtual reality videogaming group, and 2 of 5 (40%) patients in the virtual reality videogaming with Bangerter foil group gained more than 0.2 in logarithm of the minimum angle of resolution of vision. Ziak et al [21] trialed the use of the Oculus Rift virtual reality head-mounted display for dichoptic virtual reality video gaming in treating 17 adults with amblyopia, achieving an OCEBM evidence level of 4. There was a significant improvement in mean amblyopic eye visual acuity (logarithm of the minimum angle of resolution: from mean 0.58, SD 0.35 to mean 0.43, SD 0.38; *P*<.01). Mean stereoacuity also improved significantly from 263.3 s of arc to 176.7 s of arc (*P*<.01).

#### Functional Improvement for Visual Disorders

Five case series studies [20,112-115] evaluated the use of extended reality technologies to improve the function of patients with visual impairment and disorders. All reached an OCEBM evidence level of 4.

Two studies evaluated the use of digital spectacles (DSpecs) to improve mobility in a total of 43 patients with peripheral visual field deficits [20,112]. DSpecs work by capturing, relocating, and resizing video signals to fit within a patient’s visual field in real time using augmented reality technology. DSpecs enabled patients with visual field defects to have improved object identification, hand-eye coordination, and walking mobility.

Sanchez et al [113] evaluated Augmented Reality Tags for Assisting the Blind, an augmented reality system which helps the user determine the position of indoor objects by generating an audio-based representation of space. Blind participants perceived Augmented Reality Tags for Assisting the Blind to be a useful tool for assisting orientation and mobility tasks.

Tobler-Ammann et al [114] evaluated the use of virtual reality exercise games to encourage exploration of neglected space in patients with visuospatial neglect after stroke. Cognitive and spatial exploration skills trended toward improvement after the use of virtual reality exercise games and continued improving at follow-up in 5 of 7 participants. Adherence rates were high, and there were no adverse events.

Melillo et al [115] evaluated the efficacy of an augmented reality wearable improved vision system for patients with color vision deficiency. The system captures and remaps colors from the environment and displays it to the user via a head-mounted display. The system significantly improved Ishihara Vision Test scores in participants with color vision deficiency (mean score 5.8 vs 14.8, *P*=.03).

## Discussion

Although a wide range of clinically evaluated ophthalmic applications of extended reality were identified, we predominantly focused on the following domains: education, diagnostics, and therapeutics. In education, simulators demonstrated efficacy and validity in improving surgical and ophthalmoscopy skills. In diagnostics, extended reality devices demonstrated proof-of-concept in displaying ocular imaging data and validity in assessing the function of patients with glaucoma. In therapeutics, heads-up surgical systems were found to be efficacious and safe alternatives to conventional microscope surgery. The overall evidence, however, for the utility of these applications is limited. Only 8 of 87 (9%) studies had OCEBM levels of evidence of 1b, which represented randomized trials with a narrow confidence interval, while 79 of 87 (91%) studies had OCEBM levels of evidence ranging from 2b to 4 (cohort studies, case-control studies, and case series). For extended reality applications only evaluated by 1 or 2 studies, this limited evidence makes it difficult to extrapolate their utility in a wider context.

The acquisition of motor skills, as described by the Fitts–Posner 3‐Stage Theory [116] and the Dreyfus and Dreyfus model [117], progresses from an initial cognitive phase, in which learners attempt to understand, to an associative phase, in which they modify movement strategies based on feedback, to a final autonomous phase, in which motor performance is fluid. Advances in surgical simulation technology have provided options to augment or replace traditional methods without compromising training efficiency and patient safety. Although most randomized trials [38,39,45-47] showed that simulator-trained groups performed better than those who had no training at all, only 3 studies [48,49,51] drew comparisons between simulator-trained participants and participants trained with conventional methods of surgical training such as wet-lab materials and real patients. The performance of simulator-trained inexperienced surgeons was superior to that of conventionally trained inexperienced surgeons only in the laser-based procedures [48,51]. Where intraocular surgery was concerned, the performance of simulator-trained inexperienced surgeons was comparable, but not superior, to that of wet-lab trained inexperienced surgeons. Moreover, they required a longer duration to complete the procedures [49]. This could be attributed to the fact that the instruments used in wet-lab training were identical to those used in the operating room, whereas surgical simulators may not have been able to emulate all tactile and ergonomic aspects of ophthalmic surgery. This suggests a continued role for wet-lab training, at least until simulators can closely replicate the full surgical experience.

It has been shown that practicing complex motor task skills is most effective in multiple short training sessions spaced over time with variable tasks [118]. Simulation-based training allows for this, and most efficacy studies [37,39,40] demonstrate that extended reality surgical simulators were able to improve surgical performance in the specific procedure for which the participants were trained; however, the same studies found little evidence of a crossover effect—that these improvements were applicable to other surgical procedures—suggesting that simulator training is highly specific. It is possible that intense focus on solitary surgical steps can result in lack of skill development in others [37], and the transfer of surgical skills thus cannot be anticipated when planning surgical training curriculum. Nonetheless, simulation training appears to reduce complications for both surgically naive and less experienced surgeons and to improve performance for experienced ones who have had a hiatus in undertaking procedures.

Studies evaluating ophthalmoscopy simulators found that simulator training can improve both direct and indirect ophthalmoscopy skills. In comparison with surgical simulators, ophthalmoscopy simulators are not as widely adopted in ophthalmic training curricula. One possible reason lies in the nature of the simulated task. Surgery performed by inexperienced novices risks harming the patient, whereas ophthalmoscopy constitutes a minor inconvenience to a patient in terms of discomfort and time. The availability of surgical simulators with efficacy and validity may be more of a necessity than ophthalmoscopy simulators.

Validity is the cornerstone upon which educational assessments depend to be appropriately justified in their application and without which the purpose of assessments in education will have little intrinsic meaning [25]. Most validity studies on educational simulators only addressed 1 or 2 sources of validity evidence. The presence of more studies addressing all sources of validity evidence would facilitate more robust interpretation of assessment scores in ophthalmic training.

In diagnostics, visualizing ocular imaging data, such as optical coherence tomography, fundus photography, and angiography in virtual reality and augmented reality can reveal important intraocular spatial relationships [119,120] and allow for interactive exploration of imaging data to aid education, understanding of diseases, clinical assessment, and therapy. These studies [119,120] demonstrated proof-of-concept, but more studies are needed to evaluate their efficacy and accuracy for clinical use. Extended reality applications also demonstrated validity evidence and feasibility in objectively assessing functional limitation and driving performance of glaucoma and hemianopic patients. The scope of their application, however, is currently limited by the small number of studies and low number of sources of validity evidence. While extended reality was able to simulate the visual environment, it was unable to account for nonvisual cues, such as sound and touch, that patients with ophthalmic disease might rely upon in daily function.

In therapeutics, heads-up surgery allowed for better visualization, better ergonomics, and reduced endoillumination intensities than those in traditional microscope surgery without compromising outcomes. Widespread adoption of heads-up surgery, however, is limited by a few factors. First, the comfort of assistant surgeons and anesthetists has been shown to be reduced due to the positioning of the heads-up display [94,96]. Second, the learning curve of heads-up surgery has yet to be studied comprehensively. Talcott et al [91] reported that surgeons had higher ease of use with the traditional microscope than with the NGENUITY 3D visualization system, showing that the preference for heads-up surgery was not unanimous. Additional experience with heads-up displays can guide ophthalmic surgeons in transitioning from traditional microscopes to these novel systems.

It has been shown that nonstereoscopic, nonimmersive binocular treatment is a promising approach in treating children with amblyopia, with positive outcomes in amblyopic eye visual acuity and stereoacuity [121,122]. Likewise, in our review, we found that stereoscopic immersive dichoptic stimulation conferred the same benefits onto amblyopic patients with amblyopia. The 2 studies included in our review reported high adherence rates [21,111], while there have been studies on nonimmersive dichoptic stimulation reporting lower adherence rates [123,124]. Although there has been no study comparing immersive dichoptic stimulation with nonimmersive dichoptic stimulation, we postulate that immersive dichoptic stimulation can engender better patient adherence and adherence to binocular treatment. Before immersive binocular treatment can be recommended over standard binocular treatment or even over conventional occlusion therapy, additional comparative studies are needed to determine if they would be appropriate replacements or adjuncts to conventional therapy. A cost-benefit analysis would also be important, given that conventional therapy is affordable yet still efficacious.

Extended reality applications are not without adverse effects. Studies have shown that viewing of 3D displays can induce objective changes to accommodative function, convergence, refractive errors, and tear films [125-130] and subjective symptoms such as asthenopia, motion sickness, fatigue, and head or neck discomfort [131]. Techniques such as discrete viewpoint control have been shown to potentially ameliorate these adverse effects, but they are not yet widely adopted [132]. Most studies in our review did not evaluate the incidence of adverse effects induced by the extended reality set-ups. While there is growing anticipation for the adoption of extended reality, more research is needed to ascertain if these adverse effects will significantly affect the efficacy of ophthalmic applications and shape user safety guidelines.

The cost of extended reality technologies will also be a major concern for potential users. One study [133] in 2013 estimated that the EyeSi surgical simulator would save the average US ophthalmic residency program $4980 yearly in nonsupply costs based on time saved in the operating room, requiring 34 years to recoup the simulator’s cost price. Another study [134] in 2013 found that nonsupply cost savings from EyeSi use were higher in larger residency programs, but still insufficient to recoup costs at 10 years. These cost-analyses, however, do not make comparisons with conventional methods of ophthalmic surgical training. The ability of extended reality surgical simulators to simulate surgical scenarios that are otherwise impossible to replicate in a wet lab, such as posterior polar cataracts, specific clock hours of zonulysis, or a shallow anterior chamber, may represent intangible cost-savings in ophthalmic pedagogy with respect to additional time spent supervising surgeons and operating room staff, resources, and schedule. The availability of such comparisons might help to better define the role of an extended reality simulator in surgical training from the perspective of cost.

Extended reality promises utility in many areas of application by overcoming the limits of the unalterable physical environment. In ophthalmic surgical education, extended reality surgical simulators demonstrate efficacy and validity in improving surgical performance. Before surgical simulators can be considered to be a competitive alternative to traditional ophthalmic surgical training, 2 main barriers need to be addressed—cost and the need for additional high-quality comparative studies. Until these issues are addressed, surgical simulators can only play a supporting role in surgical training programs, despite their versatility and ability to provide quantitative feedback. In therapy, extended reality heads-up surgical systems have already seen popular use in ophthalmic surgery, with the literature showing that this type of system provides an efficacious and safe platform for surgical visualization. Other diagnostic and therapeutic applications mainly demonstrate proof-of-concept, with a lack of robust comparative evidence. Additional comparative studies with designs that allow a high level of evidence should be encouraged to explore the efficacy of extended reality in these varied ophthalmic applications. As extended reality is a nascent technology, we predict that it will only continue to demonstrate value and offer novel alternatives in ophthalmic education, diagnostics, and therapy.

## Appendix

Multimedia Appendix 1 Search strategy.

Multimedia Appendix 2 Messick’s five sources of validity evidence.

Multimedia Appendix 3 Studies evaluating validity of assessment based on surgical simulators.

Multimedia Appendix 4 Case-series and cohort studies evaluating the use of heads-up surgical systems.

## Abbreviations

DSpecs: digital spectacles
GRASIS: Global Rating Assessment of Skills in Intraocular Surgery
OCEBM: Oxford Centre for Evidence-Based Medicine
OSACSS: Objective Structured Assessment of Cataract Surgical Skill Score
OSATS: Objective Structured Assessment of Technical Surgical Skills
PRISMA: Preferred Reporting Items for Systematic Reviews and Meta-analyses
