# Supplementary material for: Applications of Extended Reality in Ophthalmology: Systematic Review
Source: J Med Internet Res. 2021 Aug 19;23(8):e24152. doi: 10.2196/24152 (PMC8414293; doi:10.2196/24152)
Supplement: Multimedia Appendix 1 [file jmir_v23i8e24152_app1.docx]

Multimedia Appendix 1. Search Strategy

| **Search Date** | **Database** | **Serial Number** | **Search String** | **Number of Studies** |
| --- | --- | --- | --- | --- |
| **29/02/20** | **Pubmed/MEDLINE** | #1 | ((Virtual Reality) OR (Augmented Reality) OR (Mixed Reality)) [All Fields] | - |
|  |  | #2 | Ophthalmology OR Ophthalmological OR Ophthalmic OR Eye OR Ophthalm* | - |
|  |  | #3 | #1 AND #2 | 11858 |
| **15/04/20** | **Embase** | #1 | ((Virtual Reality) OR (Augmented Reality) OR (Mixed Reality)) [All Fields] | - |
|  |  | #2 | Ophthalmology OR Ophthalmological OR Ophthalmic OR Eye OR Ophthalm* | - |
|  |  | #3 | #1 AND #2 | 622 |
| **15/04/20** | **CENTRAL** | #1 | ((Virtual Reality) OR (Augmented Reality) OR (Mixed Reality)) [All Fields] | - |
|  |  | #2 | Ophthalmology OR Ophthalmological OR Ophthalmic OR Eye OR Ophthalm* | - |
|  |  | #3 | #1 AND #2 | 135 |
|  | **TOTAL** |  |  | 12615 |
