# Supplementary material for: Applications of Extended Reality in Ophthalmology: Systematic Review
Source: J Med Internet Res. 2021 Aug 19;23(8):e24152. doi: 10.2196/24152 (PMC8414293; doi:10.2196/24152)
Supplement: Multimedia Appendix 2 [file jmir_v23i8e24152_app2.docx]

Multimedia Appendix 2. Messick’s five sources of validity evidence[1]

| **Validity Evidence Source** | **Definition** | **Examples** |
| --- | --- | --- |
| **Content** | The “relationship between a test’s content and the construct it is intended to measure.” | Test blueprint Representativeness of items to the domain  Logical/empirical relationship of content tested to achievement domain  Development strategies to ensure appropriate content representation  Item writer qualifications  Analyses by experts for adequacy of items representing the content domain |
| **Response Process** | Analyses of responses (actions, strategies, thought processes) of individual respondents or observers. Differences in response processes may reveal sources of variance irrelevant to the construct being measured. It includes instrument security, scoring, and reporting of results. | Trainee format familiarity  Understandable/accurate descriptions/interpretations of scores for trainees  Rater training Quality control of scoring Validation of preliminary scores (pilot study)  Accuracy in combining different format scores  Quality control/accuracy of final scores/marks/grades  Subscore/subscale analyses  Accuracy of applying pass–fail decision rules to scores |
| **Internal Structure** | Degree to which individual items within an instrument fit the underlying constructs. It is often reported by measures of internal consistency reliability and factor analysis | Item analysis data [item difficulty/discrimination, item/test characteristic curves (ICCs/TCCs), interitem correlations, item-total correlations]  Score scale reliability  Generalizability  Item factor analysis  Psychometric model |
| **Relationship with other variables** | Relationship between scores and other variables relevant to the construct being measured. Relationships may be positive (convergent/predictive) or negative (divergent/discriminant) depending on the constructs being measured. | Correlation with other variables or scores on other performance assessments (correlation between postgraduate level and scores)  Test-criterion correlations  Generalizability of evidence |
| **Consequences** | Assessments are intended to have some desired effect or may have unintended effects. | Impact of test scores/results on trainees  Consequences for learners/future learning  Positive consequences outweigh unintended negative consequences?  Reasonableness of method of establishing pass–fail (cut) score  Pass–fail consequences (P/F decision reliability–classification accuracy)  Instructional/learner consequences  Method of determining pass–fail score; differential pass–fail rates among examinees expected to perform similarly |

1. Ghaderi I, Manji F, Park YS, Juul D, Ott M, Harris I, et al. Technical skills assessment toolbox: a review using the unitary framework of validity. Ann Surg. 2015 Feb;261(2):251-62. PMID: 24424150. doi: 10.1097/sla.0000000000000520.
