# Supplementary material for: Applications of Extended Reality in Ophthalmology: Systematic Review
Source: J Med Internet Res. 2021 Aug 19;23(8):e24152. doi: 10.2196/24152 (PMC8414293; doi:10.2196/24152)
Supplement: Multimedia Appendix 3 [file jmir_v23i8e24152_app3.docx]

Multimedia Appendix 3: Studies evaluating validity of assessments based on surgical simulators.

| **Study** | **Surgical Simulator** | **Simulated Task (Modules)** | **Validity Evidence Addressed** |
| --- | --- | --- | --- |
| Thomsen (2017a) [39] | EyeSi® | Cataract surgery, Vitreoretinal surgery | All |
| Selvander (2012) [40] | EyeSi® | Cataract surgery (Navigation, capsulorhexis) | Content, Relationship with other variables, Response process |
| Bozkurt (2018) [43] | EyeSi® | Cataract surgery (Capsulorhexis) | Relationship with other variables |
| Sikder (2015) [52] | MicroVisTouch™ | Cataract surgery (Capsulorhexis) | Content, Relationship with other variables |
| Saleh (2013b) [53] | EyeSi® | Cataract surgery (CCC, Cracking and chopping, Cataract navigation, Bimanual cataract training, Anti-tremor) | Content, Internal Structure |
| Rossi (2004) [54] | EyeSi® | Vitreoretinal surgery (Navigation, Epiretinal membrane removal) | Content, Relationship with other variables |
| Mahr (2008) [55] | EyeSi® | Cataract Surgery (Forceps and anti-tremor) | Content, Internal Structure, Relationship with other variables |
| Solverson (2009) [56] | EyeSi® | Basic navigational microdexterity module | Content, Relationship with other variables |
| Privett (2010) [57] | EyeSi® | Cataract surgery (Capsulorhexis) | Content, Relationship with other variables, Response process |
| Nathoo (2011) [58] | EyeSi® | Anterior segment tasks (Not specified) | Relationship with other variables |
| Le (2011) [59] | EyeSi® | Cataract surgery (Forceps, anti-tremor, capsulorhexis) | Content, Relationship with other variables |
| Cisse (2019)  [60] | EyeSi® | Vitreoretinal surgery (navigation, forceps, vitrector, and epiretinal membrane peeling) | Content, Relationship with other variables, Response process |
| Spiteri (2014) [61] | EyeSi® | Cataract surgery (Forceps, anti-tremor, capsulorhexis, phaco divide and conquer) | Content, Relationship with other variables, Response process |
| Lam (2016) [62] | Phacoemulsification simulator | Cataract surgery | Content, Relationship with other variables, Response process |
| Selvander (2013b) [63] | EyeSi® | Cataract surgery (Capsulorhexis, hydromaneuvers, and phacoemulsification divide-and-conquer) | Content, Internal Structure, Relationship with other variables |
| Thomsen (2015) [64] | EyeSi® | Cataract Surgery (All except chopping) | All |
| Thomsen (2017b) [65] | EyeSi® | Cataract surgery (Intracapsular navigation, anti-tremor, intracapsular anti-tremor, forceps, bimanual, capsulorhexis and phaco divide and conquer) | Content, Relationship with other variables |
| Jacobsen (2019) [66] | EyeSi® | Cataract surgery (Intracapsular navigation, anti-tremor, intracapsular anti-tremor, forceps, bimanual, capsulorhexis and phaco divide and conquer) | Content, Relationship with other variables |
| Vergmann (2017) [67] | EyeSi® | Vitreoretinal surgery (Navigation, forceps, bimanual, laser coagulation, posterior hyaloid level and internal limiting membrane peeling) | Content, Relationship with other variables, Response process |
| Selvander (2013a) [68] | EyeSi® | Cataract surgery (Capsulorhexis, hydromaneuvers and phacoemulsification) | Content, Relationship with other variables |
