# Supplementary material for: Applications of Extended Reality in Ophthalmology: Systematic Review
Source: J Med Internet Res. 2021 Aug 19;23(8):e24152. doi: 10.2196/24152 (PMC8414293; doi:10.2196/24152)
Supplement: Multimedia Appendix 4 [file jmir_v23i8e24152_app4.docx]

Multimedia Appendix 4: Case-series and cohort studies evaluating the use of heads-up surgical systems

| **Study** | **Design (OCEBM)** | **Heads-up Surgical System** | **Surgical Procedure** | **Conventional microscope, n** | **Heads-up, n** | **Outcome Measure** |
| --- | --- | --- | --- | --- | --- | --- |
| Zhang (2019a) [94] | Case-control (4) | NGENUITY 3D® | Vitreoretinal surgeries | 28 | 31 | Endoillumination intensity, procedure time, user perception, complications |
| Matsumoto (2019) [95] | Case series (4) | Self-assembled | Cataract surgery, vitrectomy, glaucoma microdevice implant | NA | 74 | Procedure success, complications, endoillumination intensity, patient comfort |
| Rizzo (2018) [96] | Case series (4) | NGENUITY 3D® | Anterior and posterior segment surgeries | NA | 200 | Procedural success, user perception, endoillumination |
| Martinez-Toldos (2017) [97] | Case series (4) | TRENION 3D HD with HMD | Cataract surgery, macular surgery, 23G-PPV, 23G-PPV silicone oil removal | NA | 9 | Complications, user perception |
| Eckardt (2016) [98] | Case series (4) | TrueVision® 3D Surgical | Vitrectomy | NA | >400 | Procedure success, complications, general evaluation, user perception |
| Kunikata (2016) [99] | Case series (4) | MKC-700HD and CFA-3DL1 | Vitrectomy | NA | 6 | Completion of procedure, complications, user perception |
| Coppola (2017) [100] | Cohort (4) | NGENUITY 3D® | Retinal detachment surgery | 15 | 7 | Procedure success, redetachment, complications, intraocular augmented pressure, procedure time, mean endoillumination power, use of triamcinolone |
| Kita (2018) [101] | Case series (4) | NGENUITY 3D® | Vitrectomy | NA | 113 | Procedural success, endoillumination intensity, reattachment |
| Kita (2019a) [102] | Case series (4) | NGENUITY 3D® with 3D endoscope system | Vitrectomy | NA | 391 | Procedure success, complications |
| Kita (2019b) [103] | Case series (4) | NGENUITY 3D® | Scleral buckle procedures | NA | 18 | Procedure success, complications |
| Agranat (2019) [104] | Case series (4) | NGENUITY 3D® | Vitreoretinal surgeries | NA | 272 | Procedure success, complications |
| Zhang (2019b) [105] | Case-control (2b) | NGENUITY 3D® | Vitrectomy | 202 | 124 | Visual acuity, procedure success, procedure time, complications |
| Palacios (2019) [106] | Cohort (2b) | NGENUITY 3D® | Macular hole repair | 20 | 20 | Procedure success, user perception |
| Ali (2017) [107] | Case series (4) | The TIPCAM® 3D ORL endoscope | Endoscopic lacrimal surgery | NA | 15 | Procedure success, procedure time, implementation, visualization, optical performance, user perception, complications |
| Ehlers (2018) [108] | Case series (4) | Zeiss Rescan 700 Integrated Intraoperative OCT and NGENUITY 3D® | Vitrectomy | NA | 7 | Procedure success, complications, user perception |
| Freeman (2019) [109] | Case series (4) | TrueVision® 3D Surgical | NA | NA | NA | Subjective vs measured lateral resolution and depth of field, user perception |
| Bhadri (2007) [110] | Case series (4) | Digital Microsurgical Workstation | Cataract surgery, vitreoretinal surgery on porcine eyes | NA | NA | Procedure success, user perception |
